# A high-protein diet with and without strength training shows no negative effects on oxidative stress markers in older adults

**DOI:** 10.1016/j.redox.2025.103707

**Published:** 2025-05-30

**Authors:** Laura Bragagna, Lina Maqboul, Ricarda Baron, Muriel Harloff, Monika Spasova, Sahar Noori, Agnes Draxler, Bernhard Franzke, Eva-Maria Strasser, Patrick A. Zöhrer, Sandra Unterberger, Rudolf Aschauer, Barbara Wessner, Karl-Heinz Wagner

**Affiliations:** a Department of Nutritional Sciences, University of Vienna, Austria; b Research Platform Active Aging, University of Vienna, Austria; c Vienna Doctoral School for Pharmaceutical, Nutritional and Sport Sciences (PhaNuSpo), Vienna, Austria; d Department of Health Sciences Dietetics, FH Campus Wien University of Applied Sciences, Vienna, Austria; e Research Center Health Sciences, FH Campus Wien University of Applied Sciences, Vienna, Austria; f Institute of Physical Medicine and Rehabilitation, Klinik Favoriten, Vienna, Austria; g Centre for Sport Science and University Sports, University of Vienna, Austria

**Keywords:** Oxidative stress, High protein diet, Antioxidants, Aging, Strength training, Older adults

## Abstract

A long, healthy and pain-free life is the goal of an aging society. However, people become increasingly less active in old age, which can lead to sarcopenia. To counteract this development, strength training and a sufficient protein supply are essential. To investigate the effects of a high-protein diet in combination with strength training on oxidative stress markers in older adults, 116 men and women underwent a 17-week single-blind randomized control trial with 3 groups (Control CON, Recommended Protein RP and High Protein HP) as part of the NutriAging Study.

After finishing a 6-week dietary intervention, a strength training program was additionally implemented for RP and HP for the remaining study period. CON continued with their habitual protein intake throughout the study. Blood was drawn at three time points (baseline T1, week 8 T2, and after study completion T3) and analyzed for chemical blood parameters and the oxidative stress markers superoxide dismutase (SOD), glutathione-peroxidase (GSH-Px), catalase (CAT), ferric reducing ability potential (FRAP); γ-glutamyl-cysteinyl-glycine (GSH), glutathione disulfide (GSSG), unconjugated bilirubin (UCB), and malondialdehyde (MDA). The results showed a significant time effect of certain blood parameters and all measured oxidative stress markers independent of group allocation. This can be explained by seasonal changes over the study period. Urea and blood urea nitrogen (BUN) were significantly increased in HP, which could be attributed to the high protein intake, however they remained in the normal range. UCB and FRAP were significantly lower in HP, which could be due to the metabolization of the additional protein. Women in particular showed more altered oxidative stress markers as well as reduced uric acid (UA) in the HP group, suggesting lower estrogen secretion during menopause, possibly due to decreased nuclear factor erythroid 2-related factor 2 (NrF2) synthesis and subsequently impaired antioxidant defense. The strength training intervention alone showed no negative effects on blood or oxidative stress parameters. In summary, a high-protein diet along with strength training showed no major impact on oxidative stress in older adults.

## Highlights

• Strength training showed no negative effects on oxidative stress markers in older adults.
• A high protein diet increased urea and blood urea nitrogen concentration in older adults, but remained within normal range.
• Post-menopausal women were more likely to be affected by oxidative stress during a high protein diet.

## Graphical abstract

**Figure:**
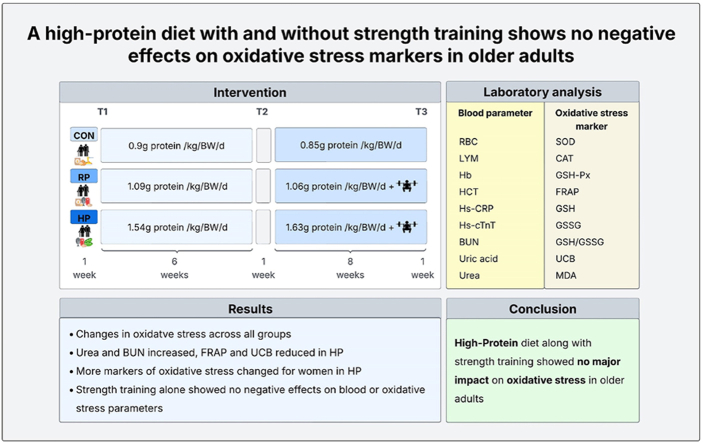

## 1 Introduction

Aging is a topic that is increasingly becoming a global matter of interest. Since maintaining a high quality of life and staying free from illness or physical complaints as long as possible is the main goal with advanced age, there are several approaches to promote healthy aging: One is physical activity, which should enable people to remain agile and mobile into old age. However, people become less physically active as they get older, which leads to age-related muscle loss resulting in reduced power, strength, skeletal muscle mass and accordingly decreased muscle quality which can result in sarcopenia. As a result, this could lead to an increased risk of sudden falls with injuries [1]. Strength training in particular can counteract this phenomenon [2]. The second approach to ensuring healthy aging is a balanced diet, with a sufficient supply of micro and macronutrients. One food group that is often inadequately supplied in old age is protein, often accompanied by a lower total energy intake [3]. The amount of protein recommended by the D-A-CH reference values for older adults 65 years or older is 1g per kilogram of body weight per day [4]. Although larger scale studies in the old (starting from 65 year) and very old (85 years and older) adults are still limited, it is apparent that protein intake below the recommended amount can lead to reduced muscle strength and physical performance due to reduced muscle protein synthesis [5]. It is therefore needed to investigate the direct impact of high-protein diets on functional markers and muscle strength of older adults [6], but equally important to observe potential negative effects on other sets of biological marker such as oxidative stress.

Aging is also accompanied by chronically elevated oxidative stress an imbalance between reactive oxygen species and their elimination by antioxidants in the body which could lead to oxidative damage to molecules such as proteins, fats and DNA [7]. In the long term, this can cause tissue damage and lead to various diseases such as coronary heart diseases or type 2 diabetes [8]. The link between a high protein diet and a potential impact on oxidative stress in elderly is under-investigated. There have been very few studies that have addressed the possible impact that higher protein intake may exert on protein oxidation, which is important for cellular processes that are redox-regulated mainly by thiol-oxidation [9]. Protein oxidation can therefore result in sustained damage of a protein structure, with the possible loss in function. In 2015, an increase in lipid oxidation and protein carbonylation during the digestion of red meat in a simulated large intestine environment was observed by Van Hecke et al. [10]. In another study, it was shown that markers for lipid and protein oxidation increased up to 25-fold the higher the iron concentration in the meat [11]. Further evidence of an increased risk of oxidative stress was shown in an in vivo study where the intake of protein after an overnight fast led to a significant increase in the formation of reactive oxygen species (ROS) in circulating white blood cells. A similar picture emerges with the intake of a very high-fat meal [12]. However, other studies carried out have not been able to experimentally demonstrate increased oxidative stress. For instance, a 15-week dietary intervention with a protein content of 51 % of total energy, carried out on adult rats, showed no increase oxidative stress levels [13]. Only a few studies have investigated the effect of a high-protein diet in combination with a resistance training program on oxidative stress. In 2019 Nabuco et al. found that 12 weeks of whey protein supplementation combined with exercise reduced plasma uric acid concentrations, but showed no effects on antioxidant enzyme activity or markers of oxidative stress in older adults [14]. Another study carried out in older people in 2013, which combined increased protein intake through supplementation with training, showed a reduction in superoxide dismutase concentrations after 12 weeks, but only in the training group, not in the protein group and also without any effect on markers for protein and lipid oxidation [15,16]. An overview of human, animal and in vitro studies investigating the effects of high protein intake on oxidative stress markers is given in Supplemental Table 1.

The aim of this secondary investigation of the NutriAging protein study was to investigate the effects of a diet with the recommended amount of protein for old adults as well as with a much higher protein intake together with strength training on oxidative stress markers. This is the first study of this size in which protein was administered mainly via whole foods and the intake was personalized. This is relevant as processed protein sources are digested differently than whole foods [17]. Hydrolyzed proteins showed accelerated absorption compared to intact food matrcies [18]. The implications of protein supplements compared to whole foods on oxidative stress are not yet clear. Some studies have discussed whether isolated protein can reduce oxidative stress [19], while others have found increased ROS levels after consumption [12]. For this reason in particular, research focusing on whole foods is needed to provide comparative data on protein supplements. Further, a whole food approach is much closer to the real-life condition than the use of protein supplements. In order to determine the level of oxidative stress in the participants over the course of the intervention, a broad spectrum of markers for oxidative stress have been considered in this 17 week lasting intervention study. These were the total antioxidant potential (FRAP), unconjugated bilirubin (UCB), reduced (GSH) and oxidized (GSSG) glutathione, malondialdehyde (MDA), as well as the antioxidant enzymes superoxide dismutase (SOD), catalase (CAT) and glutathione peroxidase (GSH-Px) - markers of oxidative stress, which have been shown to be reliable in various human studies [[20], [21], [22], [23], [24], [25]]. The combination of classic blood parameters with the oxidative stress markers mentioned shall provide an overview of the health status and the effective age of the test persons and furthermore show whether a protein-rich diet, alone and in combination with a strength training intervention, affects the state of health of older adults.

## 2 Material and methods

### 2.1 Study design and participants

**Fig. 1:**
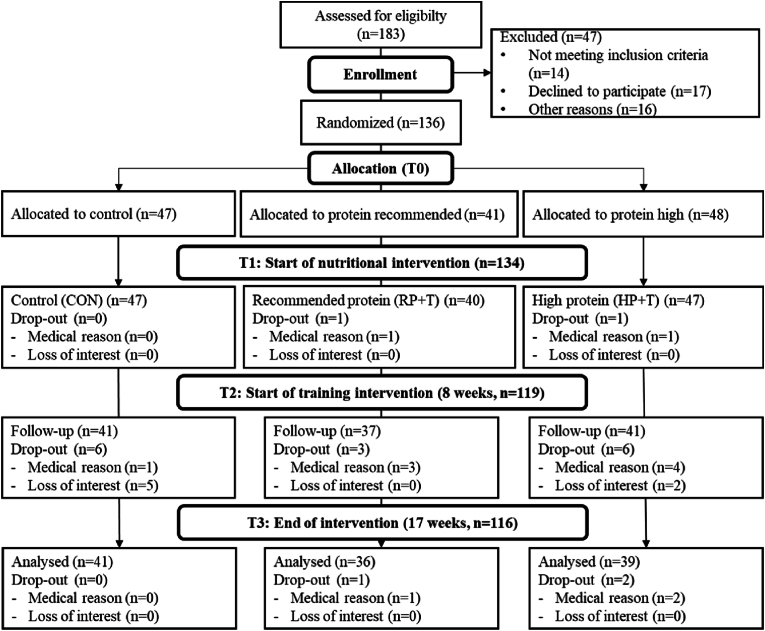
Flow diagram of the study intervention.

The present study was a randomized, single-blinded controlled trial with three groups. A control group (CON) in which the participants maintained their usual eating habits, a group where the protein intake should follow the protein recommendation for this age group (1g protein/kg/BW) (RP) and a group aiming to double that recommended protein intake (2g protein/kg/BW) (HP). The initial dietary intervention lasted 6 weeks, followed by an 8-week phase in which progressive strength training was carried out in addition to the dietary change. Data was collected at baseline (T1), after 8 weeks (T2) and after 17 weeks (T3). All measurements took place at the Centre for Sport Science and University Sports, University of Vienna, Austria between July and December 2018. To be eligible for study enrollment, participants had to be between 65 and 85 years old and not performing regular strength training 6 months prior to the study. Exclusion criteria were diseases that could be worsened by the training intervention, as well as major cognitive limitations (Mini Mental State Examination Score <23), severe cardiovascular issues, diabetic retinopathy and pronounced osteoporosis. Furthermore, the consumption of cortisone and anticoagulants as well as a frailty index of >3 or walking aids was a reason for not being able to participate in the study. A health history of cancer, coronary heart disease and diabetes was not an automatic exclusion criterion for the study, as long as it had no negative influence on the implementation of the intervention. All participants gave their written consent to participate in the study. The study was conducted according to the ethical guidelines of the Declaration of Helsinki, approved by the Ethics Committee of the University of Vienna (Reference Number: 00322) and registered under https://clinicaltrials.gov (NCT04023513). The flowchart of the study is shown in Fig. 1.

### 2.2 Nutrition and training intervention

The diet and exercise intervention has already been described in detail by Unterberger et al. [6]. Briefly, starting right after the collection of the baseline data (T1) the CON group received no nutritional intervention, while RP and HP received a personalized diet. RP received the amount recommended by the D-A-CH reference values for older adults, which is 1 g/kg/BW [26]. For HP, the goal was to double the recommended amount of protein intake. The main focus of the two intervention groups was to achieve the desired protein intake without the addition of supplements, instead working with nutritional counseling and for HP with protein-rich foods that are readily available in stores such as high-protein bread, puddings, dairy products, soups and pea protein sticks. The RP group received foods with a medium protein content such as milk products, homemade muffins, and bars in order to compensate for the energy intake by the HP group. The exact protein intake was observed, documented and calculated. Dietary intake data were assessed by 24-h recalls every 7–10 days. Over the whole study period participants completed on average 9 ± 1 interviews, out of those referred to weekdays and 2 to weekend days. To evaluate and adapt participants diets each interview was analyzed within 2 days. The first 4 interviews were face-to-face, the following interviews could also be conducted via telephone. To assist the interview process, the software Globodiet® was used [27]. Furthermore, participants received a photo book to support estimate portion size. Dietary intake data were linked to the German food composition database (Bundeslebensmittelschlüssel) version 3.02 [28] and total energy intake (kcal), carbohydrates (g/kg BW/d), fat (g/kg BW/d) and protein (g/kg BW/d) were estimated. Furthermore, participants reported the consumption of provided foods in a food diary collected during the interviews. The food provided was personalized and always delivered 2 weeks in advance. In the training phase of the study, HP received a hazelnut-chocolate or vanilla drink (Veganeo, AnovonA®) with 146 kcal and 32g protein, while RP received an isocaloric carbohydrate-rich drink (Bulkpowders.com) with 152 kcal and 0g protein, both to be consumed immediately after training. For CON, their eating behavior was monitored but not changed. The total daily energy intake was based on the D-A-CH reference values with a PAL index of 1.4-1-6. This resulted in 1700 kcal/d for women and 2100–2500 kcal/d for men.

The strength training intervention has already been described in detail by Franzke et al., 2022 [29]. In summary, the strength training took place in the period between T2 and T3 for 8 weeks. The training was conducted in commercial gyms twice a week and was carried out according to the guidelines of the American College of Sport Medicine (ACSM) [30]. The training for RP and HP was the identical, consisting of a 5–10-min warm-up followed by 45–60 min of training for all major muscle groups. The strength training comprised five machine-guided exercises (leg press/leg curl/latissimus pulldown/rowing/chest press), two free weight exercises free weight exercises (goblet box squat/shoulder press) and one bodyweight exercise (front plank with alternating leg lifts). The test subjects were instructed to remain in the concentric phase for 1–2 s and in the eccentric phase for 3–4 s. The intensity was subjectively adjusted using the OMNI scale for subjective perception of exertion (RPE 0–10). In the first two weeks, the participants underwent a familiarization phase with one to two sets of 15–20 repetitions at submaximal load (RPE 3–4). From the third week, the subjects increased the weight and reduced the number of repetitions to 10–15 repetitions for 2 sets with an RPE of 4–6. From week 6, the intensity was further increased to an RPE of 6–7, with the number of repetitions reduced to 8–12 repetitions and the number of sets increased to three. The weight was individually RPE adjusted and the load was increased if it fell below 4 or if more than 12 repetitions could be completed. Afterwards there was a cool down phase for 5–10 min. The intensity of the training was individually adapted to the subjective assessment of the participants. All training sessions were performed in small groups and supervised by sport scientists who monitored and, if necessary, corrected the correct and safe execution [6].

### 2.3 Blood sampling and sample preparation

Blood was collected at Centre for Sport Science and University Sports, University of Vienna, Austria using EDTA plasma and serum tubes (BD Vacutainer®) and then immediately transported to the Department of Nutritional Sciences at the University of Vienna, Austria where it was prepared, aliquoted and immediately frozen at −80 °C until analyses.

### 2.4 Blood chemistry

Immediately after blood collection from the volunteers, some of the collection tubes were transported to a routine medical laboratory (Dr Claudia Vidotto study lab GMBH, 1230 Vienna) and analyzed for the concentration of standard blood parameters red blood cells (RBC), lymphocytes (LYM), hemoglobin (Hb), hematocrit (HCT), high-sensitive CRP (hs-CRP), high sensitive troponin (hs-cTnT), uric acid (UA) and urea.

### 2.5 Analyses of oxidative stress markers

#### 2.5.1 Total antioxidant activity (FRAP)

To determine the total antioxidant activity, an adapted version of the FRAP assay by Benzie et al. was applied [31]. The concept of this method is a Fe3+ complex, which is reduced to Fe2+ upon interaction with antioxidants and turns from colorless to blue. This allows to determine the amount of antioxidants in the plasma spectrophotometrically. In detail, 10 μl of blank (distilled water), standard (FeSO_4_∗7H_2_O), control (Trolox) or sample (EDTA plasma) were applied to 96 well microtiter plates, diluted with 30 μl of distilled water in the plate and 300 μl of FRAP reagent (25 mL 300mmoL acetate buffer, 2.5 mL 10mmoL TPTZ and 2.5 mL 20mmoL FeCl_3_∗6H_2_O) were added. After 6 min of incubation at 37 °C, photometric measurements were taken at 595 nm, and the total antioxidant concentration of the samples was determined using the standard [32].

#### 2.5.2 Reduced (GSH) and oxidized (GSSG) glutathione

The method of Hissin and Hilf [33] was used to determine GSH and GSSG. By labeling the SH group using the fluorescent *o*-phthalaldehyde (OPA), the concentration of glutathione in human plasma samples can be determined photometrically using UV light. Plasma samples pre-treated with TCA were used in duplicate for the method. To determine GSH, 10 μL sample or standard with 180 μL 5 nM EDTA buffer (pH 8) and 10 μL 1 M OPA were applied to a 96 well microtiter plate and measured photometrically after 15min incubation in darkness on a shaker with fluorescence. For the GSSG measurement, the standards or samples were first incubated with 40 mM N-ethylmaleimide for 30min in the dark, then 10 μL of the product was mixed with 180 μL 0.1 M NaOH (pH 12) and 10 μL 1 M OPA in the microtiter plate and also incubated for 30min in the dark on the shaker and then measured fluorometrically. The concentration of the two substances in the samples was determined using the standard curves for GSH and GSSG [34].

#### 2.5.3 Unconjugated bilirubin (UCB)

The endogenous antioxidant UCB is a degradation product of hemoglobin and was determined by HPLC in EDTA plasma. The initial method previously described by Wallner et al., 2012 [35] and updated by Schoissengeier et al., 2024 [36] was used for this purpose. Briefly, 50 μL EDTA plasma were mixed with 200 μL mobile phase (96.5 % methanol) and centrifuged for 10 min at 14000 rpm and 4 °C. 120 μL supernatant was transferred to HPLC vials and transferred to the autosampler. For HPLC analysis, a Fortis C18 HPLC column (4.6 × 150 mm, 3 mm), a Phenomenex Security Guard cartridge for C18 HPLC columns (4 × 3 mm), and a photodiode array detector (PDA, Shimadzu, Tokyo, Japan) were used. The flow rate was 1 mL/min. Retention time (rt) of the IXa peak was 8–9 min. Bilirubin (purity ≥98 %, Sigma Aldrich) was used as an external standard to determine the UCB concentration (3.3 % IIIα, 92.8 % IXα, and 3.9 % XIIIα isomers, 450 nm). Two human serum samples were used as quality control (QC) and evaluated per analysis run as internal standards.

#### 2.5.4 Analysis of enzymatic antioxidants superoxide dismutase (SOD), catalase (CAT), and glutathione peroxidase (GSH-Px)

For sample preparation, red blood cells from whole blood were washed three times with isotonic buffer and then a 1:2 suspension was prepared with the buffer. Subsequently, 300 μL of the suspension were mixed with 450 μL distilled water to obtain a hemolysate.

The antioxidant enzymes SOD, GSH-Px and CAT were determined according to the established methods of Beutler and Marklund and Marklud [37,38]. Briefly, SOD activity was defined by its inhibition of the auto-oxidation of 1,2,3-trihydroxybenzene (pyrogallol) in the presence of superoxide anions (O2-), one unit of SOD activity was defined as the amount of SOD required to inhibit autoxidation by 50 %. GSH-Px activity was defined in relation to the oxidation of NADPH2 to NADP+. The activity catalyzing the oxidation of 1 nmol of NADP + per minute was defined as one unit. CAT activity was measured by the rate of degradation of hydrogen peroxide (H2O2), One unit of CAT activity was defined as the rate constant of the first-order reaction [25].

#### 2.5.5 Malondialdehyde (MDA)

As previously described by Franzke et al. [39], the MDA concentration in plasma was determined by HPLC. After heating at 100 °C for 60 min, the plasma samples were neutralized with methanol/NaOH, centrifuged (3 min, 3000 rpm) and determined at excitation: λ = 532 nm, emission: λ = 563 nm, using the LaChrom Merck Hitachi Chromatography System (Vienna, Austria) and an HPLC column 125 × 4 mm, 5 μm (Merck, Vienna, Austria).

### 2.6 Statistics

All statistical analyses were carried out using IBM SPSS Statistics 28 software. A one-factorial analysis of variance was carried out to test the baseline data. A repeated measures analysis of variance (RM-ANOVA) was performed to statistically analyze time and group effects as well as group interactions (CON, RP and HP). A Greenhouse-Geisser correction was always performed if sphericity was not present. Post-hoc tests with Bonferroni correction were then conducted to compare the individual time points. Significance was set to α = 0.05.

## 3 Results

### 3.1 Baseline characteristics

The baseline characteristics of the participants split by sex are described in Table 1. As described by Unterberger et al. [6], 116 volunteers participated in the study, 61 of them were signed at birth as female and 55 as male. The mean age was 72.8 ± 4.6 years (mean ± standard deviation), with 73.1 ± 4.4 for the females and 72.4 ± 4.8 for the males. The mean BMI of the participants of both sexes together was 26.1 ± 3.9 kg/m^2^, for the female participants 25.7 ± 4.2 and for the male participants 26.5 ± 3.63, with an overall mean waist to hip ratio of 0.9 + 0.08, with 0.86 + 0.06 for the female participants and 0.96 ± 0.06 for the male participants. The age and body composition as well as the energy intake and the intake of macro nutrients were evenly distributed in all three intervention groups at the beginning of the study, as can be seen in Table 2. The baseline blood parameters RBC, Hb, HCT, hs-cTnT and UA showed a significant difference between men and women. The baseline data of the oxidative stress markers show that there are significant differences between male and female participants for FRAP and UCB (Table 1).

**Table 1:** Baseline characteristics of the participants divided by sex.

Parameter	Women		Men		Total		
	61		55		116		
	MW±	SD	MW±	SD	MW±	SD	p-value
Age [years]	73.09±	4.41	72.39±	4.83	72.76±	4.60	0.411
BMI [kg/m^2^]	25.74±	4.19	26.45±	3.63	26.08±	3.93	0.326
Waist to Hip Ratio	0.86±	0.06	0.96±	0.06	0.90±	0.08	**<.001**
Lean body mass [kg]	47.70±	5.86	66.97±	7.50	56.38±	11.69	**<.001**
Extra cellular mass [kg]	25.61±	3.43	34.53±	4.78	29.63±	6.04	**<.001**
Body fat [kg]	19.60±	7.62	15.60±	5.73	17.80±	7.09	**0.003**
Body fat [%]	28.25±	6.29	18.56±	4.97	23.88±	7.49	**<.001**
Energy intake [kcal/d]	1604±	623	2112±	702	1847±	707	**<.001**
Protein intake [g/kg BW/d]	0.78±	0.37	0.90±	0.30	0.84±	0.34	0.059
Carbohydrates [g/kg BW/d]	2.61±	1.40	2.65±	1.05	2.63±	1.24	0.846
Fat intake [g/kg BW/d]	1.05±	0.50	1.09±	0.50	1.07±	0.50	0.719
RBC [T/L]	4.43±	0.28	4.73±	0.38	4.57±	0.36	**<.001**
LYM [%]	30.90±	7.66	28.82±	6.63	29.91±	7.24	0.122
Hb [g/dL]	13.36±	0.77	14.90±	1.18	14.09±	1.25	**<.001**
HCT [%]	39.00±	2.42	42.78±	3.22	40.79±	3.39	**<.001**
Hs-CRP [mg/L]	2.32±	2.22	2.10±	2.79	2.21±	2.50	0.638
Hs-cTnT [ng/L]	3.88±	1.89	4.78±	2.06	4.31±	2.02	**0.015**
BUN [mg/dL]	16.03±	5.77	17.22±	4.12	16.59±	5.07	0.21
UA [mg/dL]	5.03±	1.26	5.92±	1.19	5.45±	1.30	**<.001**
Urea [mg/dL]	34.36±	12.30	36.93±	8.79	35.58±	10.81	0.203
SOD [I.U./g Hb]	2175±	265.1	2139±	182.6	2154±	220.0	0.431
CAT [I.U./g Hb]	30.23±	7.35	30.55±	6.01	30.38±	6.72	0.798
GSH-Px [I.U./g Hb]	31.69±	6.85	29.98±	6.00	30.78±	6.43	0.18
FRAP [μmol/L]	1139±	186.2	1235±	176.8	1184±	187.2	**0.006**
GSH [μmol/L]	16.14±	3.46	17.19±	4.00	16.64±	3.75	0.136
GSSG [μmol/L]	8.14±	1.39	8.65±	1.43	8.38±	1.43	0.056
GSH/GSSG Ratio	2.00±	0.33	2.00±	0.36	2.00±	0.34	0.961
UCB [μmol/L]	4.85±	1.79	6.66±	3.21	5.71±	2.71	**<.001**
MDA [μmol/L]	2.22±	0.47	2.15±	0.49	2.19±	0.45	.467

Data are presented as mean ± standard deviation, the p-values were determined using a one-way ANOVA. A p-value of 0.05 is considered significant between sex. RBC: Red blood cells, Hb: Hemoglobin, HCT: Hematocrit, LYM: Lymphocytes, hs-CRP: High sensitivity c-reactive protein, UA: Uric acid, hs-cTnT: Hs-troponin, BUN: Blood urea nitrogen SOD: Superoxide dismutase, GSH-Px: Glutathione-Peroxidase, CAT: Catalase, FRAP: Ferric reducing ability potential; GSH: γ-glutamyl-cysteinyl-glycine; GSSG: Glutathione disulfide; UCB: Unconjugated bilirubin; MDA: Malondialdehyde.

**Table 2:** Baseline characteristics of the participants divided by intervention groups.

Parameter	CON		RP		HP		Total		
	41		36		39		116		
	MW±	SD	MW±	SD	MW±	SD	MW±	SD	p-value
Age [years]	72.69±	4.61	72.42±	4.31	73.14±	4.94	72.76±	4.60	0.795
BMI [kg/m^2^]	25.98±	3.94	26.36±	4.32	25.91±	3.63	26.08±	3.93	0.871
Waist to Hip Ratio	0.91±	0.07	0.89±	0.08	0.91±	0.08	0.90±	0.08	0.34
Lean body mass [kg]	55.79±	11.07	56.90±	12.90	56.55±	11.51	56.38±	11.69	0.917
Extra cellular mass [kg]	29.25±	5.96	29.68±	6.38	29.99±	5.96	29.63±	6.04	0.866
Body fat [kg]	18.11±	6.86	18.39±	7.85	16.96±	6.74	17.80±	7.09	0.659
Body fat [%]	24.43±	7.87	24.30±	7.41	22.95±	7.24	23.88±	7.49	0.64
Energy intake [kcal/d]	1838±	726	1962±	691	1752±	704	1847±	707	0.445
Protein intake [g/kg BW/d]	0.83±	0.40	0.89±	0.28	0.80±	0.32	0.84±	0.34	0.534
Carbohydrates [g/kg BW/d]	2.68±	1.38	2.71±	1.12	2.50±	1.21	2.63±	1.24	0.725
Fat intake [g/kg BW/d]	1.08±	0.56	1.08±	0.40	1.04±	0.53	1.07±	0.50	0.914

Data are presented as mean ± standard deviation, the p-values were determined using a one-way ANOVA. A p-value of 0.05 is considered significant.

### 3.2 Nutritional intervention

As can be seen in Table 3, the nutritional intervention showed that the increase in protein intake was successful. While the CON group kept their protein intake constant, the RP group was able to increase their intake to 1.06 g/kg/BW. The HP group did not reach the goal of 2 g/kg/BW, however they were able to double their initial protein intake up to 1.63 g/kg. Carbohydrate and fat intake did not change significantly. Between T1 and T2 energy intake was significantly increased in all three groups over the course of the study.

**Table 3:** Macronutrient intake over the course of the study (CON, RP, HP).

		T1		T2		T3		p-value		
Parameters	Group	Mean±	SD	Mean±	SD	Mean±	SD	Time	Time x Group	Group
Energy intake [kcal/d]	CON	1838±	726.1	1961±	578.0	1898±	528.2	**0.002**	0.693	0.334
	RP	1962±	690.9	2115±	639.4	2107±	565.7			
	HP	1752±	703.5	2002±	509.6	1975±	497.9			
	Total	1847±	706.8	2022±	574.3	1988±	532.3			
Protein intake [g/kg BW/d]	CON	0.83±	0.40	0.90±	0.36	0.85±	0.26	**<.001**	**<.001**	**<.001**
	RP	0.89±	0.28	1.09±	0.33	1.06±	0.26			
	HP	0.80±	0.32	1.54±	0.36	1.63±	0.37			
	Total	0.84±	0.34	1.18±	0.44	1.18±	0.45			
Carbohydrate intake [g/kg BW/d]	CON	2.68±	1.38	2.70±	1.09	2.82±	1.06	0.74	0.664	0.338
	RP	2.71±	1.12	2.84±	0.96	2.74±	0.76			
	HP	2.50±	1.21	2.52±	0.88	2.41±	0.89			
	Total	2.63±	1.24	2.68±	0.98	2.66±	0.93			
Fat intake [g/kg BW/d]	CON	1.08±	0.56	1.18±	0.44	1.06±	0.39	0.138	0.878	0.901
	RP	1.08±	0.40	1.13±	0.37	1.05±	0.34			
	HP	1.04±	0.53	1.11±	0.38	1.07±	0.35			
	Total	1.07±	0.50	1.14±	0.40	1.06±	0.36			

Data are presented as mean ± standard deviation, the p-values were determined using a repeated measures ANOVA. A p-value of 0.05 is considered significant.

### 3.3 Blood parameters

Blood parameters were determined at all three blood sampling times and compared to see if they had undergone any significant changes (Table 4). Bonferroni adjusted post hoc analyzes in both sexes together showed that RBC, BUN and urea increased constantly over the period of the three time points, while UA was reduced. HCT and hs-cTnT increased between the measurement times T1 and T2 but then decreased again. For BUN and urea, significant differences were also found between the three protein groups CON, RP and HP. For both markers the values very significantly increased in the HP group.

**Table 4:** Blood parameters at different time points for both sexes (CON, RP, HP).

		T1		T2		T3		p-value		
Parameters	Group	Mean±	SD	Mean±	SD	Mean±	SD	Time	Time x Group	Group
RBC [T/L]	CON	4.54±	0.42	4.58±	0.39	4.61±	0.43	**<.001**	0.716	0.672
	RP	4.61±	0.29	4.66±	0.31	4.67±	0.31			
	HP	4.57±	0.37	4.65±	0.36	4.63±	0.38			
	Total	4.57±	0.36	4.63±	0.36	4.64±	0.37			
Hb [g/dL]	CON	13.98±	1.35	13.97±	1.32	14.02±	1.35	0.689	0.658	0.58
	RP	14.23±	1.11	14.28±	1.07	14.29±	1.03			
	HP	14.07±	1.28	14.16±	1.19	14.04±	1.16			
	Total	14.09±	1.25	14.13±	1.20	14.11±	1.19			
HCT [%]	CON	40.32±	3.84	41.05±	3.56	41.24±	3.72	**<.001**	0.485	0.257
	RP	41.42±	2.73	42.42±	2.67	42.36±	2.79			
	HP	40.72±	3.44	41.97±	3.35	41.46±	3.37			
	Total	40.79±	3.39	41.78±	3.26	41.66±	3.34			
LYM [%]	CON	29.88±	7.72	30.32±	7.99	30.83±	7.36	0.161	0.968	0.727
	RP	30.61±	7.18	31.47±	8.31	31.22±	7.94			
	HP	29.31±	6.89	29.92±	6.42	30.28±	6.93			
	Total	29.91±	7.24	30.54±	7.57	30.77±	7.35			
Hs-CRP [mg/L]	CON	1.48±	1.47	2.62±	6.79	1.62±	1.15	0.397	0.437	0.208
	RP	3.17±	3.23	2.82±	2.68	3.18±	5.20			
	HP	2.11±	2.35	3.14±	5.19	3.72±	7.57			
	Total	2.21±	2.50	2.86±	5.22	2.81±	5.33			
UA [mg/dL]	CON	5.52±	1.36	5.49±	1.28	5.48±	1.19	**<.001**	**<.001**	0.091
	RP	5.31±	1.05	5.13±	1.08	5.07±	1.13			
	HP	5.51±	1.45	4.64±	1.22	4.60±	1.21			
	Total	5.45±	1.30	5.09±	1.24	5.06±	1.23			
Hs-cTnT [ng/L]	CON	4.23±	1.98	4.72±	3.25	4.10±	3.54	**<.001**	0.504	0.876
	RP	4.24±	1.96	4.39±	2.42	3.64±	2.11			
	HP	4.45±	2.15	4.47±	2.13	3.96±	1.82			
	Total	4.31±	2.02	4.53±	2.64	3.91±	2.62			
BUN [mg/dL]	CON	17.20±	6.20	16.20±	4.18	16.61±	5.20	**<.001**	**<.001**	**.012**
	RP	16.39±	3.67	16.69±	3.92	17.19±	3.93			
	HP	16.15±	4.93	20.64±	5.34	20.59±	4.92			
	Total	16.59±	5.07	17.84±	4.92	18.13±	5.02			
Urea [mg/dL]	CON	36.88±	13.20	34.71±	8.93	35.56±	11.11	**<.001**	**<.001**	**0.012**
	RP	35.11±	7.80	35.72±	8.37	36.83±	8.50			
	HP	34.64±	10.56	44.15±	11.53	44.08±	10.50			
	Total	35.58±	10.81	38.20±	10.55	38.82±	10.76			

Data are presented as mean ± standard deviation, the p-values were determined using a repeated measures ANOVA. A p-value of 0.05 is considered significant. RBC: Red blood cells, Hb: Hemoglobin, HCT: Hematocrit, LYM: Lymphocytes, hs-CRP: High sensitivity c-reactive protein, UA: Uric acid, hs-cTnT: Hs-troponin, BUN: Blood urea nitrogen.

A similar picture emerged in the female cohort. RBC, BUN and urea increased constantly over the course of the study, while UA was reduced. For hs-cTnT and HCT, as in the overall group, the parameters also increased at time point T2 but then decreased again. Only in the female group UA was significantly different between the three protein groups. Less parameters were significantly changed in the male cohort, but those showed the same pattern as for females. BUN increased consistently over the course of the study, while UA decreased. The parameters HCT and urea increased at time point T2 and then decreased again. In the male participants, significant differences were again found between the three protein groups for BUN and urea, where the HP group showed the highest concentrations. The results are summarized in Table 5, Table 6.

**Table 5:** Blood parameters at different time points for females (CON, RP, HP).

		T1		T2		T3		p-value		
Blood parameters	Group	Mean±	SD	Mean±	SD	Mean±	SD	Time	Time x Group	Group
RBC [T/L]	CON	4.42±	0.32	4.48±	0.28	4.50±	0.31	**0.004**	0.989	0.684
	RP	4.48±	0.20	4.52±	0.25	4.53±	0.22			
	HP	4.39±	0.31	4.46±	0.29	4.46±	0.32			
	Total	4.43±	0.28	4.49±	0.27	4.50±	0.29			
Hb [g/dL]	CON	13.36±	0.80	13.39±	0.68	13.43±	0.62	0.54	0.977	0.171
	RP	13.57±	0.64	13.59±	0.54	13.65±	0.53			
	HP	13.16±	0.84	13.26±	0.76	13.22±	0.82			
	Total	13.36±	0.77	13.41±	0.67	13.43±	0.68			
HCT [%]	CON	38.64±	2.57	39.68±	2.23	39.86±	2.15	**<.001**	0.873	**0.036**
	RP	40.05±	1.78	41.11±	1.82	41.05±	1.75			
	HP	38.40±	2.54	39.65±	2.74	39.25±	2.67			
	Total	39.00±	2.42	40.11±	2.36	40.03±	2.31			
LYM [%]	CON	32.23±	7.37	32.68±	8.42	33.95±	6.02	0.101	0.796	0.355
	RP	31.74±	8.37	32.42±	9.26	32.79±	7.69			
	HP	28.65±	7.13	30.85±	6.68	30.45±	7.37			
	Total	30.90±	7.66	32.00±	8.09	32.44±	7.06			
Hs-CRP [mg/L]	CON	1.74±	1.88	3.60±	9.23	1.61±	1.13	0.585	0.434	0.761
	RP	2.63±	2.03	3.08±	2.79	2.36±	1.58			
	HP	2.66±	2.67	2.51±	2.00	3.91±	8.43			
	Total	2.32±	2.22	3.08±	5.80	2.60±	4.96			
UA [mg/dL]	CON	5.31±	1.60	5.22±	1.31	5.22±	1.30	**<.001**	**0.001**	**0.013**
	RP	4.96±	0.85	4.69±	0.79	4.63±	0.83			
	HP	4.78±	1.14	4.05±	0.93	3.90±	0.95			
	Total	5.03±	1.26	4.67±	1.14	4.61±	1.18			
Hs-cTnT [ng/L]	CON	3.59±	1.11	3.79±	1.01	2.90±	0.93	**<.001**	0.075	0.544
	RP	4.02±	2.22	4.35±	2.73	3.11±	2.10			
	HP	4.06±	2.26	4.19±	2.32	3.86±	1.97			
	Total	3.88±	1.89	4.10±	2.09	3.28±	1.74			
BUN [mg/dL]	CON	17.00±	7.77	15.32±	4.44	17.41±	6.62	**0.004**	**0.01**	0.432
	RP	15.47±	3.19	16.16±	4.30	17.32±	4.27			
	HP	15.50±	5.22	19.25±	4.94	19.45±	5.33			
	Total	16.03±	5.77	16.87±	4.81	18.05±	5.55			
Urea [mg/dL]	CON	36.41±	16.52	32.86±	9.52	37.27±	14.15	**0.005**	**0.012**	0.434
	RP	33.16±	6.80	34.63±	9.21	37.05±	9.28			
	HP	33.25±	11.19	41.15±	10.70	41.65±	11.32			
	Total	34.36±	12.30	36.13±	10.32	38.64±	11.87			

Data are presented as mean ± standard deviation, the p-values were determined using a repeated measures ANOVA. A p-value of 0.05 is considered significant. RBC: Red blood cells, Hb: Hemoglobin, HCT: Hematocrit, LYM: Lymphocytes, hs-CRP: High sensitivity c-reactive protein, UA: Uric acid, hs-cTnT: Hs-troponin, BUN: Blood urea nitrogen.

**Table 6:** Blood parameters at different time points for males (CON, RP, HP).

		T1		T2		T3		p-value		
Blood parameters	Group	Mean±	SD	Mean±	SD	Mean±	SD	Time	Time x Group	Group
RBC [T/L]	CON	4.68±	0.49	4.69±	0.48	4.74±	0.51	0.054	0.638	0.616
	RP	4.76±	0.31	4.82±	0.32	4.82±	0.32			
	HP	4.77±	0.32	4.86±	0.31	4.81±	0.36			
	Total	4.73±	0.38	4.79±	0.38	4.79±	0.40			
Hb [g/dL]	CON	14.69±	1.51	14.63±	1.57	14.71±	1.63	0.715	0.606	0.578
	RP	14.97±	1.07	15.06±	0.99	15.01±	0.99			
	HP	15.04±	0.89	15.11±	0.73	14.90±	0.78			
	Total	14.90±	1.18	14.93±	1.16	14.87±	1.18			
HCT [%]	CON	42.26±	4.20	42.63±	4.18	42.84±	4.51	**0.002**	0.561	0.447
	RP	42.94±	2.84	43.88±	2.74	43.82±	3.05			
	HP	43.16±	2.43	44.42±	1.87	43.79±	2.30			
	Total	42.78±	3.22	43.64±	3.13	43.47±	3.39			
LYM [%]	CON	27.16±	7.38	27.58±	6.65	27.21±	7.23	0.965	0.475	0.432
	RP	29.35±	5.55	30.41±	7.23	29.47±	8.08			
	HP	30.00±	6.75	28.95±	6.16	30.11±	6.62			
	Total	28.82±	6.63	28.93±	6.65	28.91±	7.28			
Hs-CRP [mg/L]	CON	1.17±	0.71	1.49±	0.74	1.62±	1.22	0.406	0.328	0.18
	RP	3.77±	4.17	2.52±	2.61	4.09±	7.39			
	HP	1.53±	1.85	3.81±	7.20	3.51±	6.77			
	Total	2.10±	2.79	2.61±	4.52	3.04±	5.75			
UA [mg/dL]	CON	5.76±	1.01	5.81±	1.21	5.78±	1.01	**<.001**	**<.001**	0.875
	RP	5.69±	1.14	5.61±	1.16	5.56±	1.23			
	HP	6.28±	1.37	5.26±	1.19	5.33±	1.02			
	Total	5.92±	1.19	5.56±	1.19	5.56±	1.08			
Hs-cTnT [ng/L]	CON	4.98±	2.49	5.81±	4.47	5.48±	4.80	0.247	0.227	0.448
	RP	4.48±	1.66	4.43±	2.11	4.24±	2.01			
	HP	4.86±	2.00	4.76±	1.93	4.07±	1.71			
	Total	4.78±	2.06	5.02±	3.09	4.61±	3.20			
BUN [mg/dL]	CON	17.42±	3.85	17.21±	3.71	15.68±	2.69	**0.011**	**<.001**	**0.005**
	RP	17.41±	4.00	17.29±	3.48	17.06±	3.63			
	HP	16.84±	4.66	22.11±	5.49	21.79±	4.26			
	Total	17.22±	4.12	18.93±	4.86	18.22±	4.42			
Urea [mg/dL]	CON	37.42±	8.26	36.84±	7.92	33.58±	5.74	**0.014**	**<.001**	**0.005**
	RP	37.29±	8.45	36.94±	7.41	36.59±	7.83			
	HP	36.11±	9.94	47.32±	11.80	46.63±	9.17			
	Total	36.93±	8.79	40.49±	10.42	39.02±	9.48			

Data are presented as mean ± standard deviation, the p-values were determined using a repeated measures ANOVA. A p-value of 0.05 is considered significant. RBC: Red blood cells, Hb: Hemoglobin, HCT: Hematocrit, LYM: Lymphocytes, hs-CRP: High sensitivity c-reactive protein, UA: Uric acid, hs-cTnT: Hs-troponin, BUN: Blood urea nitrogen.

### 3.4 Oxidative stress markers

Considering the markers for oxidative stress in both sexes together, each of these markers changed over the period of the study (Table 7). The antioxidant enzymes SOD, CAT and GSH-Px increased over the three measurement times, while the non-enzymatic markers for oxidative stress FRAP and UCB were reduced, with the exception of GSH, GSSG and MDA which only dropped between T1 and T2 and then increased again at T3. In addition, a significant effect between the three groups CON, RP and HP was found in UCB and FRAP. Both markers were the lowest in the HP group.

**Table 7:** Oxidative stress markers at different timepoints for both sexes (CON, RP, HP).

		T1		T2		T3		p-value		
Parameters	Group	Mean±	SD	Mean±	SD	Mean±	SD	Time	Time x Group	Group
SOD [I.U./g Hb]	CON	2128±	212.2	2184±	251.1	2251±	284.8	**<.001**	0.451	0.07
	RP	2120±	174.1	2095±	247.2	2177±	218.5			
	HP	2214±	256.3	2263±	271.5	2312±	239.2			
	total	2154±	220.0	2185±	262.7	2250±	255.3			
GSH-Px [I.U./g Hb]	CON	31.56±	7.34	32.31±	8.08	32.08±	8.16	**0.026**	0.782	0.582
	RP	29.71±	5.85	30.04±	6.59	30.75±	5.91			
	HP	30.52±	6.23	31.15±	7.14	31.27±	7.47			
	Total	30.68±	6.55	31.28±	7.35	31.43±	7.29			
CAT [I.U./g Hb]	CON	30.17±	6.74	30.57±	7.16	30.48±	7.40	**0.029**	0.197	0.679
	RP	31.40±	7.41	31.94±	8.08	31.56±	7.25			
	HP	29.67±	6.07	30.35±	6.36	31.17±	6.99			
	Total	30.38±	6.72	30.92±	7.18	31.04±	7.17			
FRAP [μmol/L]	CON	1198±	204.1	1185±	189.4	1164±	171.2	**<.001**	**<.001**	**0.039**
	RP	1168±	171.6	1141±	155.5	1143±	155.9			
	HP	1186±	188.5	1066±	168.4	1027±	168.3			
	Total	1185±	188.0	1132±	178.2	1112±	174.9			
GSH [μmol/L]	CON	15.98±	3.71	15.58±	2.69	16.28±	3.15	**0.003**	0.265	0.322
	RP	17.49±	3.63	16.17±	2.92	17.01±	2.84			
	HP	16.57±	3.85	15.86±	2.36	15.96±	3.08			
	Total	16.64±	3.75	15.85±	2.64	16.39±	3.04			
GSSG [μmol/L]	CON	8.23±	1.47	8.17±	1.46	8.68±	1.24	**0.01**	0.071	0.902
	RP	8.38±	1.36	8.17±	1.50	8.74±	1.39			
	HP	8.54±	1.45	8.17±	1.08	8.21±	1.20			
	Total	8.38±	1.43	8.17±	1.35	8.54±	1.29			
GSH/GSSG Ratio	CON	1.95±	0.34	1.95±	0.37	1.89±	0.32	0.122	0.61	0.306
	RP	2.09±	0.28	2.02±	0.39	1.97±	0.33			
	HP	1.96±	0.38	1.96±	0.29	1.96±	0.32			
	Total	2.00±	0.34	1.97±	0.35	1.94±	0.32			
UCB [μmol/L]	CON	6.07±	2.81	6.52±	3.56	6.12±	3.74	**0.014**	0.096	**<.001**
	RP	6.55±	3.21	5.88±	2.46	5.69±	2.29			
	HP	4.57±	1.50	4.65±	1.67	3.76±	1.32			
	Total	5.72±	2.71	5.70±	2.80	5.21±	2.86			
MDA [μmol/L]	CON	2.19±	0.50	1.44±	0.33	1.68±	0.37	**<.001**	**.007**	.736
	RP	2.11±	0.43	1.35±	0.28	1.88±	0.44			
	HP	2.24±	0.51	1.56±	0.52	1.64±	0.31			
	Total	2.18±	0.48	1.45±	0.40	1.73±	0.38			

Data are presented as mean ± standard deviation, the p-values were determined using repeated measures ANOVA. A p-value of 0.05 is considered significant. SOD: Superoxide dismutase, GSH-Px: Glutathione-peroxidase, CAT: Catalase, FRAP: Ferric reducing ability potential; GSH: γ-glutamyl-cysteinyl-glycine; GSSG: Glutathione disulfide; UCB: Unconjugated bilirubin, MDA: Malondialdehyde.

Dividing the participants by sex shows that there were significant changes in the majority of markers for women over the course of the study (Table 8). SOD increased steadily, while FRAP decreased. However, GSH-Px only increased between T1 and T2 and then decreased at T3, while GSH, GSSG and MDA decreased between T1 and T2 and then increased again. A significant difference between the three protein groups was also found in the female participants for UCB and FRAP, with the lowest concentrations in the HP group. As can be seen in Table 9, the male participants showed fewer significant changes in the oxidative stress markers. Only SOD, FRAP, GSH and MDA changed significantly over the course of the study. While SOD increased steadily, FRAP decreased. GSH and MDA decreased between T1 and T2 and then increased at T3. Differences between the three protein groups were found for UCB, in the HP group the concentration was significantly lower.

**Table 8:** Oxidative stress markers at different timepoints for females (CON, RP, HP).

		T1		T2		T3		p-value		
Parameters	Group	Mean±	SD	Mean±	SD	Mean±	SD	Time	Time x Group	Group
SOD [I.U./g Hb]	CON	2120±	252.7	2204±	263.0	2258±	311.6	**0.025**	0.466	0.232
	RP	2175±	138.0	2151±	302.8	2190±	238.4			
	HP	2254±	350.6	2375±	342.8	2388±	272.4			
	Total	2175±	265.1	2243±	305.4	2281±	286.1			
GSH-Px [I.U./g Hb]	CON	33.08±	8.17	34.52±	9.33	34.19±	8.77	**0.014**	0.722	0.394
	RP	28.87±	5.92	29.94±	6.10	30.70±	4.93			
	HP	32.02±	6.65	33.47±	6.94	32.65±	8.03			
	Total	31.68±	7.22	33.02±	7.94	32.82±	7.67			
CAT [I.U./g Hb]	CON	29.12±	6.71	29.34±	6.93	29.48±	6.96	0.082	0.693	0.536
	RP	31.33±	8.84	32.18±	9.61	31.99±	8.71			
	HP	30.41±	6.59	30.88±	6.04	31.75±	6.88			
	Total	30.23±	7.35	30.73±	7.60	31.00±	7.50			
FRAP [μmol/L]	CON	1185±	224.3	1139±	197.3	1139±	181.0	**<.001**	0.125	**0.011**
	RP	1131±	163.7	1083±	129.8	1107±	119.5			
	HP	1095±	159.5	988.2±	146.3	950.6±	169.1			
	Total	1139±	187.8	1073±	171.7	1069±	177.5			
GSH [μmol/L]	CON	15.08±	3.35	14.81±	2.07	15.80±	2.60	**0.05**	0.062	0.144
	RP	17.62±	3.74	15.82±	3.34	16.95±	3.10			
	HP	15.97±	3.00	15.74±	1.93	15.34±	2.24			
	Total	16.14±	3.46	15.42±	2.48	15.99±	2.69			
GSSG [μmol/L]	CON	8.06±	1.44	7.78±	1.69	8.55±	1.43	**0.026**	0.223	0.925
	RP	8.12±	1.49	7.56±	1.68	8.29±	1.41			
	HP	8.24±	1.32	8.07±	1.09	7.99±	0.96			
	Total	8.14±	1.39	7.81±	1.50	8.29±	1.28			
GSH/GSSG Ratio	CON	1.88±	0.30	1.97±	0.42	1.88±	0.35	0.257	0.797	0.06
	RP	2.17±	0.27	2.15±	0.47	2.07±	0.35			
	HP	1.97±	0.36	1.98±	0.29	1.94±	0.31			
	Total	2.00±	0.33	2.03±	0.40	1.96±	0.34			
UCB [μmol/L]	CON	5.17±	1.86	5.89±	2.86	4.98±	2.39	**0.005**	0.388	**0.028**
	RP	5.26±	1.98	5.01±	1.66	4.74±	1.50			
	HP	4.12±	1.30	4.38±	1.79	3.40±	0.91			
	Total	4.87±	1.79	5.13±	2.27	4.40±	1.86			
MDA [μmol/L]	CON	2.30±	0.45	1.48±	0.32	1.71±	0.28	**<.001**	.093	.895
	RP	2.19±	0.36	1.35±	0.33	1.84±	0.40			
	HP	2.13±	0.57	1.62±	0.63	1.65±	0.33			
	Total	2.21±	0.47	1.48±	0.46	1.73±	0.34			

Data are presented as mean ± standard deviation, the p-values were determined using repeated measures ANOVA. A p-value of 0.05 is considered significant. SOD: Superoxide dismutase, GSH-Px: Glutathione-peroxidase, CAT: Catalase, FRAP: Ferric reducing ability potential; GSH: γ-glutamyl-cysteinyl-glycine; GSSG: Glutathione disulfide; UCB: Unconjugated bilirubin, MDA: Malondialdehyde.

**Table 9:** Oxidative stress markers at different timepoints for males (CON, RP, HP).

		T1		T2		T3		p-value		
Parameters	Group	Mean±	SD	Mean±	SD	Mean±	SD	Time	Time x Group	Group
SOD [I.U./g Hb]	CON	2135±	175.3	2166±	245.8	2244±	267.1	**<.001**	0.88	0.213
	RP	2088±	188.7	2062±	211.3	2169±	213.1			
	HP	2189±	180.7	2193±	193.5	2263±	208.9			
	Total	2139±	182.6	2143±	221.3	2227±	231.1			
GSH-Px [I.U./g Hb]	CON	30.20±	6.43	30.34±	6.39	30.19±	7.29	0.413	0.841	0.974
	RP	30.20±	5.93	30.09±	7.04	30.77±	6.57			
	HP	29.57±	5.93	29.69±	7.05	30.40±	7.19			
	Total	29.98±	6.00	30.04±	6.70	30.44±	6.91			
CAT [I.U./g Hb]	CON	31.38±	6.74	31.99±	7.34	31.64±	7.91	0.31	0.28	0.626
	RP	31.49±	5.54	31.64±	6.07	31.05±	5.24			
	HP	28.93±	5.58	29.83±	6.79	30.60±	7.25			
	Total	30.55±	6.01	31.13±	6.73	31.10±	6.86			
FRAP [μmol/L]	CON	1213±	182.9	1239±	169.1	1193±	158.8	**0.002**	**0.003**	0.683
	RP	1211±	175.1	1206±	159.3	1183±	184.0			
	HP	1277±	173.6	1143±	155.9	1104±	131.4			
	Total	1235±	176.8	1196±	163.8	1159±	160.7			
GSH [μmol/L]	CON	17.01±	3.91	16.47±	3.09	16.83±	3.69	**0.043**	0.951	0.935
	RP	17.36±	3.62	16.54±	2.45	17.07±	2.64			
	HP	17.20±	4.58	15.99±	2.79	16.62±	3.72			
	Total	17.19±	4.00	16.32±	2.76	16.83±	3.35			
GSSG [μmol/L]	CON	8.43±	1.53	8.63±	1.01	8.82±	1.01	0.262	0.087	0.557
	RP	8.65±	1.20	8.81±	0.95	9.22±	1.23			
	HP	8.86±	1.55	8.28±	1.10	8.45±	1.41			
	Total	8.65±	1.43	8.56±	1.03	8.82±	1.24			
GSH/GSSG Ratio	CON	2.03±	0.37	1.91±	0.31	1.90±	0.28	0.069	0.522	0.893
	RP	2.00±	0.27	1.88±	0.21	1.87±	0.28			
	HP	1.96±	0.42	1.94±	0.29	1.98±	0.34			
	Total	2.00±	0.36	1.91±	0.27	1.92±	0.30			
UCB [μmol/L]	CON	7.11±	3.37	7.26±	4.19	7.44±	4.58	0.216	0.261	**0.007**
	RP	7.99±	3.74	6.86±	2.87	6.77±	2.57			
	HP	5.01±	1.57	4.92±	1.55	4.11±	1.58			
	Total	6.66±	3.21	6.33±	3.19	6.08±	3.46			
MDA [μmol/L]	CON	2.06±	0.53	1.39±	0.34	1.64±	0.46	**<.001**	**.016**	.340
	RP	2.02±	0.48	1.35±	0.21	1.92±	0.48			
	HP	2.36±	0.41	1.50±	0.39	1.63±	0.30			
	Total	2.15±	0.49	1.42±	0.33	1.72±	0.43			

Data are presented as mean ± standard deviation, the p-values were determined using repeated measures ANOVA. A p-value of 0.05 is considered significant. SOD: Superoxide dismutase, GSH-Px: Glutathione-peroxidase, CAT: Catalase, FRAP: Ferric reducing ability potential; GSH: γ-glutamyl-cysteinyl-glycine; GSSG: Glutathione disulfide; UCB: Unconjugated bilirubin, MDA: Malondialdehyde.

## 4 Discussion

The aim of the secondary analysis of the NutriAging study was to investigate whether a high-protein diet, alone and in combination with resistance training, affects blood parameters and oxidative stress markers in older adults. Baseline data were collected to ensure that the participants had the same starting conditions. Furthermore, food intake was closely monitored by repeated 24 recalls across the study period, and energy and macro nutrient intake were analyzed. As already described by Franzke et al., the CON group was able to keep their protein intake constant at 0.8–0.9 g/kg/BW/d. The dietary intervention allowed the participants in the RP group to initially increase and subsequently maintain their protein intake to the recommended level of 1 g/kg/BW/d for their age group, and the HP group managed to double their initial protein intake from 0.8 g/kg/BW/d to 1.6 g/kg/BW/d^29^.

The additional protein intake was facilitated via nutritional counseling and commercially available protein-rich foods such protein-rich milk products, bars, puddings, protein-rich bread, bacon crisps, protein rich soups, pea protein sticks as well as recipes for self-prepared foods. These protein sources were derived from both animal and plant origin. However, since participants were free to also select from the offered options, it is not possible to determine the exact ratio between plant-based to animal-based protein consumed.

Protein quality plays a critical role, particularly for muscle protein synthesis in older adults, as well as in promoting other associated biological effects. Given the diverse range of protein sources provided, each with varying amino acid profiles, we assume a high overall protein quality. This is likely due to the complementary effects of combining different protein sources, which help to compensate for limiting amino acids in individual foods.

We also monitored the intake of antioxidative vitamins and minerals and found no significant differences between the groups. However, it was not possible to account for the intake from the provided food sources, as detailed information on their micronutrient content was not available on the packaging and was not provided by the producers.

For all participants BUN and urea increased in the HP group. Even though these changes were physiologically of minor relevance, they were significant. Urea is traditionally a marker for kidney disease, there is also increasing evidence that urea itself triggers molecular changes associated with insulin resistance, free radical production and apoptosis [40]. The same applies to BUN which uses nitrogen equivalents as the unit for the urea mass concentration instead of the complete urea molecule. However, increased BUN values are very typical with a high-protein diet and are not automatically a sign of possible illnesses [41]. In this study in particular BUN levels with a mean of 20.59 ± 4.92 mg/dL were still within the reference range for older adults of 8–28 mg/dL [42]. Nevertheless, it is worth noting that some theories suggest that chronically high circulating BUN levels could lead to oxidative stress, inflammation, endothelial dysfunction, and cardiovascular disease as well as type II diabetes by enhancing protein carbamylation and generating reactive oxygen species [[43], [44], [45]]. Another measured safety marker to control kidney health throughout the study was the glomerular filtration rate (GFR). After the HP intervention, the participants' GFR values of 73.93 + 11.27 was still within the recommended range of >60 mL/min [46].

Only in the female group UA was significantly different between the three protein groups. The significantly lower concentration of UA in the HP group could have various reasons. On the one hand, this could indicate higher oxidative stress in that group, as UA is not only a degradation product of purine nucleosides and free bases, but also acts as an antioxidant in plasma [47]. On the other hand, high levels of UA are linked to hypertension, visceral obesity, insulin resistance, diabetes type II and many other lifestyle-associated diseases [[48], [49], [50], [51]]. A reduction could therefore also indicate the positive effects of a high-protein diet in combination with exercise. It is therefore not clear whether this reduction in UA levels would have a long-term positive or negative effect on the female participants. The very low changes in the blood parameters over the course of the study in men could indicate that women react more sensitively to the strength training intervention.

A time effect was observed for all measured oxidative stress markers over the course of the study, regardless of the protein intervention. Although these effects were minor, they were significant. It has already been established that oxidative stress can subsequently accelerate aging processes, but with increasing age, antioxidant defense is impaired and the accumulation of ROS leads to post-transcriptional changes that can be used as biomarkers for oxidative stress [52]. Aging is associated with strength deficits such as frailty which can lead to sarcopenia. ROS also contribute to sarcopenia by increasing proteolysis and impair muscle protein synthesis, leading to a reduction in muscle mass [53]. Furthermore, ROS cause a change in actin and myosin structures that significantly reduce the cross-bridge cycle in the myofibrillar apparatus [54]. However regarding the time effect specifically, as already discussed by Draxler et al., one possible reason could be the seasonal changes over the period of the study [16]. The enrollment into the study and the associated investigations were conducted from July to the end of August 2018. During this period, Austria experienced heatwaves, with 29 out of 48 days in which the temperature exceeded 30 °C and 45 out of 48 days recorded temperatures above 25 °C. The second investigations took place in the period from September to October, when a temperature of over 30 °C was only measured on 3 days and only 16 over 25 °C. Bhat et al. found increased erythrocyte thiobarbituric acid reactive substances (TBARS) and plasma cortisol levels as well as decreased erythrocyte reduced glutathione levels, erythrocyte glutathione peroxidase and superoxide dismutase activities in rats during the hot season compared to the cool season in India [55]. During the summer period increased ozone levels were also detected in Austria. Chen et al. found increased lipid peroxidation in healthy young adults with elevated ozone levels in summer in California [56]. Rossner Jr. et al. also observed increased lipid oxidation in city bus drivers during the summer months with high ozone levels [57].

A significant difference between the three protein groups was also found in the female participants for UCB and FRAP, in the male group however only for UCB. A possible reason for the reduction in the two antioxidant markers UCB and FRAP could be the increased production of ROS due to a higher demand for NADH in the body which is produced during the breakdown of amino acids as part of transamination and oxidative deamination. Furthermore, throughout this process, nitrogen is eliminated in the form of ammonia via the urea cycle, which can indirectly increase oxidative stress due to the increased energy required. In this way, more antioxidants may be needed to compensate for the imbalance [58]. Other possible reasons for a decrease in those two oxidative stress markers measured in the HP group could be enhanced ammonia levels due to triggered protein metabolism, which may subsequently lead to an increase in ROS via the urea cycle. In vitro and animal model studies on urea cycle disorders (UCD) suggest that not only the accumulation of ammonia, but also of the other metabolites involved in UCD can influence redox status [59]. Other studies on hyperammonemia in cell, animal or human models have also shown mitochondrial dysfunction and oxidative stress. This suggests that the role of ammonia is crucial in the generation of oxidative stress [60]. Other studies showed that increased BCAA concentrations induce ROS generation and mitochondrial dysfunction in PBMCs. These pro-oxidative and pro-inflammatory mechanisms of BCAA are mediated by the Akt-mTORC1 axis [61]. This suggests that BCAAs could influence the cellular stress state via mTORC1, which could indirectly affect the activity of antioxidant enzymes. It is also worth noting that the majority of antioxidants measured in the FRAP method consist of UA [31]. This overlaps with the previously discussed reduction in the UA measured in the HP group.

The baseline data already showed that the female participants had significantly lower concentrations of the antioxidant markers FRAP and UCB than males (Table 4). This can be explained by higher oxidative stress to which women are exposed from the menopause onwards due to the lower concentrations of estradiols. In particular, 17β-estradiol, estriol, estrone, ethinylestradiol and 2-hidroxiestradiol have an antioxidant effect due to an intact hydroxyl group on ring A of the molecule [[62], [63], [64]]. This results in a reduction of nuclear factor erythroid 2-related factor 2 (Nrf2), which is a key transcription factor of the oxidative stress response [65,66]. Sindan et al. have previously shown that in female mice NrF2 is predominantly produced in the granulosa cells and oocytes of the secondary and antral follicles of the ovaries [67]. Subsequently, this age-related decrease in Nrf2 in women leads to a reduced antioxidant capacity and impaired protection against oxidative stress [68,69]. The study also showed that markers for oxidative stress such as GSH-Px, GSSG and UCB were significantly more altered in the female participants than in the male participants. Previous data has already found a highly significant reduction in glutathione levels in postmenopausal women, as well as SOD, CAT, and MDA which could be due to an increased need for ROS defense due to higher levels of oxidative stress [70,71]. This effect was amplified by the study intervention. In the female participants, it was shown that UCB and FRAP were significantly lower in the CON group than in the HP group, while the male participants did not show significantly lower FRAP values. This indicates that the female participants reacted more sensitively to the high protein in the diet then men. However, it is worth noting that both UCB and FRAP are indirect markers of oxidative stress. The direct marker for oxidative stress MDA remained unchanged between the three protein groups. The possible reasons for the lower concentration of antioxidants in the HP protein groups described above have so far only been investigated in mixed-sex cohorts and not in postmenopausal women alone. These results are particularly interesting as most previous studies have only found the positive effects of higher protein intake on body composition and muscle growth in postmenopausal women [72,73]. However, the effects of a high-protein diet on oxidative stress markers in postmenopausal women have remained relatively unexplored.

None of the measured markers, neither the blood parameters nor those for oxidative stress, showed any negative effects of resistance training. Hs-cTnT, a very specific and sensitive marker of myocardial damage [74], decreased between T2 and T3, indicating that moderate strength training does not have a negative effect on heart health and may even be protective. HCT also decreased between T2 and T3. Previous studies have shown that regular exercise can significantly reduce hematocrit, suggesting that regular physical activity improves blood flow properties and may have positive effects on cardiovascular health [75].

The study had many strengths but also some limitations. Particularly notable as a strength was the food-based approach to protein supply, as most studies focusing on high-protein diets used supplements. The original goal of getting the HP group to 2 g/kg BW/d was not achieved, but the original protein intake was doubled to 1.6 g/kg BW/d. With 116 participants and an equal distribution between men and women, the entire group of elderly people could be well reflected. In addition to the nutritional intervention, an exercise intervention was also successfully carried out. All measured oxidative stress markers could also be measured within our own laboratories, which ensured a constant quality over the whole period of the study. The heat waves during the summer seem to have triggered increased oxidative stress in the participants and were reflected in the measured markers. In a country with constant temperatures all year round, this factor could be more easily eliminated. This was also shown in the paper by Draxler et al., in which no time effect on DNA damage or glutathione was found in samples from New Zealand, where the temperature never exceeded 25° during the study period [16]. We also attempted to measure a comprehensive set of oxidative stress biomarkers and the activity of oxidative stress defense enzymes. However, we acknowledge that additional markers, both direct and indirect, are available and could provide further insights. Dietary intake was assessed using 24-h recalls, which have inherent limitations in capturing high-quality intake data. To address this limitation, we collected an average of nine recalls per participant over the study period. With this frequency, we are confident that the dietary intake data obtained is reliable.

## 5 Conclusion

The aim of this study was to investigate the effects of a high-protein diet alone and together with strength training on biochemical markers of oxidative stress and aging. The food-based dietary intervention was successful and the participants in the HP group were able to double their baseline protein intake. The training intervention was also successfully carried out. A total of 116 older adults were able to complete this study. The examination of the blood parameters revealed a time effect in most of the measured markers and a significant increase in urea and BUN in the HP group compared to the other intervention groups. The differences were not meaningful and still within the range of reference values, showing that the protein intervention was well tolerated. Only for the female participants UA significantly decreased in the HP group, which could be interpreted in two ways since UA is an antioxidant as well as a marker for multiple diseases.

Looking at dynamics of oxidative stress, there was a time effect in all measured markers. When they were separated by sex, a clear picture emerged showing a higher exposure of female participants to oxidative stress. This can be explained by the low estrogen levels triggered by the menopause, as estrogen is known to act as an antioxidant. Some of the observed effects can partly be explained by severe weather fluctuations over the course of the study. Hot temperatures, high ozone levels and strong sunlight exposure can have effects on oxidative stress levels in humans.

In summary, a high-protein diet combined with moderate resistance training was well tolerated and did not lead to negative effects on oxidative stress in older individuals.

## CRediT authorship contribution statement

**Laura Bragagna:** Writing – original draft, Visualization, Validation, Investigation. **Lina Maqboul:** Investigation. **Ricarda Baron:** Investigation. **Muriel Harloff:** Investigation, Conceptualization. **Monika Spasova:** Investigation. **Sahar Noori:** Investigation. **Agnes Draxler:** Writing – review & editing, Investigation. **Bernhard Franzke:** Writing – review & editing, Methodology, Investigation, Conceptualization. **Eva-Maria Strasser:** Writing – review & editing, Methodology, Conceptualization. **Patrick A. Zöhrer:** Investigation. **Sandra Unterberger:** Investigation, Data curation. **Rudolf Aschauer:** Writing – review & editing, Investigation. **Barbara Wessner:** Resources, Funding acquisition, Formal analysis, Conceptualization. **Karl-Heinz Wagner:** Writing – review & editing, Supervision, Resources, Project administration, Methodology, Funding acquisition, Conceptualization.

## Declaration of generative AI and AI-assisted technologies in the writing process

During the preparation of this work the author used the translation software deepL SE as part of this body of work in order to improve the English wording. After using this tool/service, the author reviewed and edited the content as needed and takes full responsibility for the content of the publication.

## Funding

This work was supported by the 10.13039/100013276EU-program Interreg SK-AT (NutriAging) and the 10.13039/501100003065University of Vienna, by funding the Research Platform Active Aging. This article is supported by the Open Access Publishing Fund of the 10.13039/501100003065University of Vienna.

## Declaration of competing interest

The authors declare that they have no known competing financial interests or personal relationships that could have appeared to influence the work reported in this paper.

## Data Availability

The data that has been used is confidential.
