# Supplementary material for: A high-protein diet with and without strength training shows no negative effects on oxidative stress markers in older adults
Source: Redox Biol. 2025 May 30;85:103707. doi: 10.1016/j.redox.2025.103707 (PMC12219370; doi:10.1016/j.redox.2025.103707)
Supplement: Multimedia component 1 [file mmc1.docx]

**Supplementary File:**

| **Supplemental Table 1: Overview of important in vitro, animal and human studies focusing on effects of high-protein diets on oxidative stress markers** | | | | | |
| --- | --- | --- | --- | --- | --- |
| **Study** | **Model** | **Age** | **Duration & Protein** | **Oxidative Stress Markers** | **Key Findings** |
| Petzke et al. (2000)^1^ | Rat | Adult | 15 weeks; casein-based diets with 13.8 % (AP), 25.7 % (MP) and 51.3 % (HP) crude protein (+ HP-tocopherol) | TBARS, GSH, LeuOX, QLeu | Long-term high-protein intake did not increase oxidative stress markers. |
| Mohanty et al. (2002)^2^ | Human (leukocytes) | 28–65 years | Single protein challenge (~1257 kJ pure casein; energy-matched to fat challenge) | ROS (PMNs & MNCs), TBARS | Both lipid and protein intake stimulated a transient increase in ROS and lipid peroxidation. |
| Shahar et al (2013)^3^ | Human | 60-74 years | 12 weeks. high protein diet (soy protein drink, 1.5 g/kg/day) vs. control vs. exercise group vs. combined exercise + high protein diet | SOD, PC, LOOH | Increased SOD in high protein diet group, no effects on PC and LOOH in any group |
| Van Hecke et al. (2014)^4^ | In vitro digestion | n/a | Simulated digestion of chicken (~1 % protein), pork (~5 %), beef (~8 %) | MDA, 4-HNE, PC | Higher heme-iron content led to more lipid and protein oxidation during digestion. |
| Moirón et al. (2015)^5^ | Rat | Adult | 12 weeks; high-protein diet (≈ 52 % protein) vs. control diet (18 % protein) | TBARS, PC, SOD, CAT | High-protein diet induced oxidative stress in rat brain; high-intensity exercise reduced TBARS. |
| Van Hecke et al. (2015)^6^ | In vitro digestion | n/a | Roasted, cured pork samples (~20 % protein): raw, cooked (65 °C/15 min), over-cooked (90 °C/30 min) | MDA, 4-HNE, PC | Over-cooking cured meat dramatically increased oxidative and nitrosative reactions. |
| Zebrowska et al. (2019)^7^ | Rat | Adult | 8 weeks; high-protein diet (44 % protein) vs. control (24.2 %) | GSH-Px, GR, CAT, SOD, GSH, UA, AGE, 4-HNE, MDA | High-protein diet induced oxidative stress in cerebral cortex and hypothalamus. |
| Zhu et al. (2017)^8^ | Rat | Young rats | 14 days; 20 % protein diets (casein, soy, fish, chicken, pork, beef) | Grx1, Trx1, GSH, SOD, MDA | Meat proteins raised ROS and TAC but lowered MDA compared to non-meat proteins. |
| Pivovarova-Ramich et al. (2020)^9^ | Human (clinical trial) | 64.3 ± 1.0 years | 6 weeks; isocaloric diets with 30 % energy from protein (animal vs. plant) | MDA, PC, NT | Both animal and plant high-protein diets improved oxidative stress markers but increased nitro-OS markers. |
| AGE: Advanced Glycation End Products, CAT: Catalase, SOD: Superoxide Dismutase, GSH-Px: Glutathione peroxidase, GR: Glutathione Reductase, GSH: γ-glutamyl-cysteinyl-glycine, Grx1: Glutaredoxin 1, LeuOX: Leucine Oxidation Rate, LOOH: Lipid hydroperoxide MDA: Malondialdehyde, MNCs: Mononuclear Cells, NT: Nitrotyrosine, OS: Oxidative Stress, PC: Protein Carbonyls, PMNs: Polymorphonuclear Leukocytes, QLeu: Leucine Flux, ROS: Reactive Oxygen Species, SOD: Superoxide Dismutase, TBARS: Thiobarbituric Acid Reactive Substances, TAC: Total Antioxidant Capacity, Trx1: Thioredoxin 1, UA: Uric Acid | | | | | |

**References**

1. Petzke, K. J., Elsner, A., Proll, J., Thielecke, F. & Metges, C. C. Long-term high protein intake does not increase oxidative stress in rats. *J. Nutr.* **130**, 2889–2896 (2000).

2. Mohanty, P. *et al.* Both lipid and protein intakes stimulate increased generation of reactive oxygen species by polymorphonuclear leukocytes and mononuclear cells. *Am. J. Clin. Nutr.* **75**, 767–772 (2002).

3. Shahar, S. *et al.* Effectiveness of exercise and protein supplementation intervention on body composition, functional fitness, and oxidative stress among elderly Malays with sarcopenia. *Clin. Interv. Aging* **8**, 1365–1375 (2013).

4. Van Hecke, T. *et al.* Nitrite curing of chicken, pork, and beef inhibits oxidation but does not affect N-nitroso compound (NOC)-specific DNA adduct formation during in vitro digestion. *J. Agric. Food Chem.* **62**, 1980–1988 (2014).

5. Camiletti-Móiron, D. *et al.* High-protein diet induces oxidative stress in rat brain: protective action of high-intensity exercise against lipid peroxidation. *Nutr. Hosp.* **31**, 866–874 (2015).

6. Van Hecke, T. *et al.* Increased oxidative and nitrosative reactions during digestion could contribute to the association between well-done red meat consumption and colorectal cancer. *Food Chem.* **187**, 29–36 (2015).

7. Żebrowska, E., Maciejczyk, M., Żendzian-Piotrowska, M., Zalewska, A. & Chabowski, A. High protein diet induces oxidative stress in rat cerebral cortex and hypothalamus. *Int. J. Mol. Sci.* **20**, (2019).

8. Zhu, J. *et al.* Oxidative and anti-oxidative status in muscle of young rats in response to six protein diets. *Sci. Rep.* **7**, 1–10 (2017).

9. Pivovarova-Ramich, O. *et al.* Effects of diets high in animal or plant protein on oxidative stress in individuals with type 2 diabetes: A randomized clinical trial: High protein diet and oxidative stress. *Redox Biol.* **29**, (2020).
